# Utilizing Integrated Bioinformatics Analysis to Explore Potential Alterations in Mitochondrial Function Within Immune Cells Associated with Thoracic Aortic Aneurysms

**DOI:** 10.3390/bioengineering12020197

**Published:** 2025-02-17

**Authors:** Chang Guan, Si-Xu Chen, Chun-Ling Huang, Yi-Peng Du, Kai-Hao Wang, Pei-Xin Li, Shen-Rong Liu, Zhao-Yu Liu, Zheng Huang

**Affiliations:** 1 Department of Cardiology, The First Affiliated Hospital of Guangzhou Medical University, Guangzhou 510120, China; guanch@gzhmu.edu.cn (C.G.); duyipeng@foxmail.com (Y.-P.D.); kaihao60@126.com (K.-H.W.); 13380595998@163.com (P.-X.L.); liushenrong0713@126.com (S.-R.L.); 2 Medical Research Center, Guangdong Provincial Key Laboratory of Malignant Tumor Epigenetics and Gene Regulation, Sun Yat-Sen Memorial Hospital, Sun Yat-Sen University, Guangzhou 510120, China; chensx@mail2.sysu.edu.cn (S.-X.C.); tina19003@163.com (C.-L.H.)

**Keywords:** thoracic aortic aneurysm (TAA), immune cells, mitochondrial dysfunction, machine learning

## Abstract

Thoracic aortic aneurysm (TAA) is a life-threatening peripheral vascular disease with a complex pathogenesis. Altered mitochondrial function in vascular smooth muscle cells has been implicated in TAA development. However, the link between mitochondrial functional status and immune cell behavior in TAA patients remains largely unexplored. In this study, we analyzed several bulk RNA-seq and snRNA-seq datasets of TAA from the NCBI-GEO and Genome Sequence Archive database, identifying differentially expressed mitochondrial-related genes (DE-MRGs). To assess mitochondrial function, we calculated a mitoscore to represent the overall expression level of MRGs. Our analysis revealed mitochondrial-mediated apoptosis occurring in M1 macrophages, while CD4 + T cells demonstrated the activation of quality control mechanisms, such as mitochondrial fission. Through LASSO regression and SVM-RFE, we identified key MRGs, including *MUCB*, *ARRB2*, *FRG*, and *ALPL*, which we further validated using TAA mouse models. Additionally, we found that DE-MRGs were closely linked to methionine metabolism. In conclusion, this study highlights mitochondrial dysfunction in immune cells associated with TAA, shedding light on potential mitochondrial roles in TAA pathogenesis.

## 1. Introduction

Thoracic aortic aneurysm is a life-threatening disease that results from locally or diffusely pathological dilation of the aorta. It is usually asymptomatic until incidentally discovered on imaging examinations or until rupture occurs [1,2]. Currently, the understanding and mechanism of TAA remain elusive, and early diagnosis and effective treatment of TAA is still a challenge in clinical practice [3,4]. The etiologies underlying TAA are diverse and can range from degenerative or hypertension-associated aortic enlargement to genetic disorders [5,6]. The pathophysiology of TAA is considered a combination of complex mechanisms, including inflammation, the proteolytic degradation of elastic tissue, the degeneration of the extracellular matrix, and the apoptosis of smooth muscle cells. Additionally, chronic inflammation of the arterial wall, caused by infiltration of various immune cells, is a core aspect of the pathogenesis [7,8]. Therefore, interfering with immune cells may provide innovative therapeutic targets for TAA.

Mitochondria, the energy factories of all cells, are dynamic organelles that undergo fission and fusion events to maintain their homeostasis. When mitochondrial dysfunction occurs at the cellular level, it affects the systemic metabolic balance and contributes significantly to many diseases. Mitochondrial dysfunction has been consistently linked to vascular aging and various cardiovascular disorders, including atherosclerosis and heart failure [9,10,11]. Recent studies have proven that the mitochondrial function of vascular smooth muscle cells (VSMCs) is controlled by the extracellular matrix and drives the development of aortic aneurysms in Marfan syndrome [12]. In non-hereditary TAA, changes in mitochondrial function and metabolism in VSMCs have also been found to contribute to disease progression [13,14]. However, it is unknown whether immune cells within the aorta exhibit mitochondrial dysfunction and play a role in the progression of TAA. Thus, exploring underlying mitochondria-related alterations of immune cells in TAA patients may provide novel insights.

With the advancement of high-throughput sequencing technology, we can detect transcriptome changes in tissues and different cells and further explore the molecular mechanisms in various diseases. In this study, using bulk RNA-seq and snRNA-seq from public databases, we investigated the altered mitochondrial function of immune cells in TAA by single-sample gene set enrichment analysis (ssGSEA). Two machine learning algorithms (SVM-RFE and LASSO) were further used to identify key mitochondria-related genes in the disease model. The results may provide a better understanding of the pathogenesis of TAA from a new perspective.

## 2. Materials and Methods

### 2.1. Data Acquisition

The transcriptome profiles and corresponding clinical information of TAA were obtained from the Genome Sequence Archive (https://bigd.big.ac.cn/gsa/, accessed on 14 November 2022) and the Gene Expression Omnibus (GEO, https://www.ncbi.nlm.nih.gov/gds/). In the present study, the transcriptome profiles from 8 TAA specimens and 6 thoracic aortic specimens were downloaded from a previous article [15]. Single-nuclear RNA-seq (snRNA-seq) data of TAA from GSE207784 datasets were also obtained from the GEO database [16]. GSE207784 included 6 TAA and 7 control thoracic aorta specimens, totaling 71,689 cells. A total of 1434 mitochondrial-related genes (MRGs) were identified in the MsigDB database (https://www.gsea-msigdb.org/gsea/msigdb/). The metabolic gene set (metabolism) for this analysis came from the GeneCard database (https://www.genecards.org/).

### 2.2. Screening Differentially Expressed Mitochondrial-Related Genes (DE-MRGs) Between TAA and Control

Limma 3.54.0 version package in R was used to screen the differentially expressed genes (DEGs) between TAA and the control specimens with the criterion of |FC| > 1.5 and a *p*-value < 0.05. Then, the DE-MRGs were obtained by overlapping the DEGs and 1434 MRGs from the MsigDB database. Subsequently, the most significant enrichment analysis of DE-MRGs was investigated using the Database for Annotation, Visualization, and Integrated Discovery (DAVID). A false discovery rate (FDR) < 0.05 was considered statistically significant.

### 2.3. snRNA-seq Data Processing

To ensure the quality of the data, the Seurat version 4.1.0 package in R was used to perform the quality control. The data were filtered based on the following criteria: total number of unique genes per cell less than 100 or more than 4000 and mitochondrial genes accounting for less than 4% in TAA. A total of 28,101 genes and 71,623 cells in TAA were selected for subsequent analysis. After normalization, 2000 highly variable genes (HVGs) were identified, and dimensionality reduction was performed through principal component analysis (PCA). The significant principal components (PCs) were identified using FindNeighbors and were then clustered using uniform manifold approximation and projection (UMAP) through FindClusters. The marker genes were obtained from CellMarker2.0 (http://117.50.127.228/CellMarker/CellMarkerBrowse.jsp/, accessed on 5 February 2025), and then the DEGs of each cell cluster were identified using FindAllMarkers functions. Finally, cell type identification was performed based on the DEGs in each cluster and manually checked according to the previous studies.

### 2.4. Evaluation of the Mitoscore

The mitoscore is a mitochondrial score that reflects the overall expression level of mitochondrial-related genes in the sample and provides quantitative indicators to describe mitochondrial function. Mitochondrial-related genes were obtained from the MsigDB database and were used as a background set to calculate the mitochondrial fraction in each sample using the ssGSEA method of the GSVA software package (1.38.2) based on bulk RNA-seq data and snRNA-seq data. The PercentageFeatureSet function in the Seurat package was employed to evaluate the expression of the MRGs. Then, the mitoscore of each cell was visualized by mapping it onto the UMAP embedding to identify active clusters. The DE-MRGs between TAA and the control specimens were identified using the Wilcoxon rank-sum test, with thresholds of |FC| > 1.5 and *p*-value < 0.05.

### 2.5. Construction of the LASSO Model and the SVM-RFE Feature Selection Process

Intersected MRGs in TAA were obtained by overlapping the DE-MRGs from bulk RNA-seq and snRNA-seq analyses, respectively. Afterward, candidate diagnostic genes were selected using two machine learning algorithms: least absolute shrinkage and selection operator (LASSO)-penalized Cox regression and support vector machine recursive feature elimination (SVM-RFE). The LASSO regression analysis was performed using the glmnet package in R to identify the MRGs that were significantly associated with the discrimination of TAA and the control specimens. The support vector machine (SVM) model is a powerful tool for identifying predictive models and classifiers, and recursive feature elimination (RFE) is the gold standard of the wrapper method for selecting optimal genes by avoiding overfitting. Finally, the overlapping genes between the two algorithms were obtained and used as the candidate genes for the diagnosis of TAA.

### 2.6. ROC Curve Analysis

The receiver operating characteristic curve (ROC) was drawn using the pROC package in R, and the area under the ROC curve (AUC) values were applied to assess the diagnostic effectiveness in discriminating TAA from the control specimens.

### 2.7. Animal Studies

The animal experiments were approved by the Laboratory Animal Ethics Committee of the Affiliated First Hospital of Guangzhou Medical University. Male C57BL/6J were purchased from Guangdong Medical Laboratory Animal Center and housed under specific pathogen-free conditions with a 12 h light/12 h dark cycle at 24 ± 2 °C and 50–70% humidity in the Laboratory Animal Center of the Affiliated First Hospital of Guangzhou Medical University.

The TAA mouse model was constructed in the following manner [4,17]. Briefly, 3-week-old male C57BL/6J mice were fed a normal diet and administered beta-aminopropionitrile (BAPN) (#A1314; Sigma-Aldrich, St. Louis, MO, USA) dissolved in their drinking water at a concentration of 1 g/kg/day for 4 weeks. The mice were weighed daily, and any that died prematurely were autopsied immediately. The surviving mice were euthanized on day 28 using an overdose of sodium pentobarbital, and the aortas were collected for further experiments. Aortic aneurysm is defined as a localized dilation of the aorta > 50% of its adjacent intake portion. Aortic aneurysm tissue was harvested, snap frozen, and stored at −80 °C for mRNA analysis.

### 2.8. qRT-PCR Analysis

The total RNA was lysed with RNAiso Plus reagent (Takara, Tokyo, Japan) and isolated following the manufacturer’s instructions. The concentration and purity of the total RNA solution were quantified using NanoDrop-2000 (Thermo Fisher Scientific, Waltham, MA, USA). The isolated RNA was reverse-transcribed to cDNA using a PrimeScript RT Reagent Kit (TaKaRa, Shiga, Japan). After this, the cDNAs were subjected to SYBR green dye-based qRT-PCR analysis. GAPDH was used as the internal control, and the relative expression of the target genes was determined using the 2^−ΔΔCt^ method. The primer sequences used in the study are shown in Table S4. All primers were synthesized by Servicebio (Servicebio, Wuhan, China).

### 2.9. Statistical Analysis

All data analyses were performed using R software (version 3.6.3), and appropriate R packages were selected. *p* < 0.05 was considered to indicate a significant statistical difference. Three independent experiments were conducted to obtain values with mean ± standard deviation. When multiple comparisons were made, a one-way analysis of variance with Bonferroni’s post-test was used; when pair-wise comparisons were made, the Student’s *t*-test was used. Pearson’s correlation test was used for correlation analyses. Statistically significant differences were defined as those with a *p*-value < 0.05.

## 3. Results

### 3.1. Bulk RNA-seq Summarized the DE-MRGs in TAA

**Figure 1:**
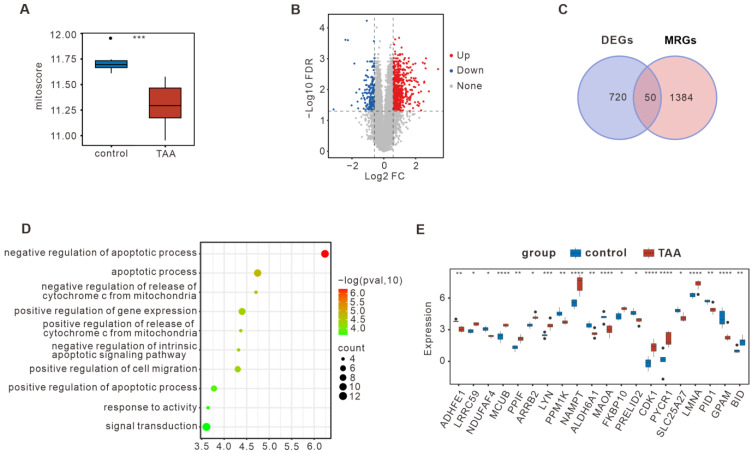
Bulk RNA-seq summarized the DE-MRGs in TAA. (**A**) Boxplots of the mitoscore between the TAA and control groups based on bulk RNA-seq. (**B**) Volcano map of the DEGs in TAA. (**C**) Venn plots of the overlapped DE-MRGs in TAA by overlapping the DEGs and MRGs from the MsigDB database. (**D**) Top 10 enriched items in the GO enrichment analysis of biological processes for the DE-MRGs in TAA. The size of the circles represents the number of genes enriched. (**E**) Boxplots of the top 20 DE-MRGs in TAA. * *p* < 0.05; ** *p* < 0.01; *** *p* < 0.001; **** *p* < 0.0001.

Human bulk RNA-seq datasets for TAA [15] were collected from public repositories. For more comprehensive information, the DEGs were screened based on |FC| > 1.5 and *p* < 0.05. To determine mitochondrial dysfunction, the mitoscore of each sample was assessed. We found a decreased mitcoscore in TAA compared to the corresponding controls (Figure 1A). As shown in the volcano plot in Figure 1B, a total of 770 DEGs (604 upregulated and 166 downregulated) were screened in TAA compared to the normal group (Table S1). To identify the DE-MRGs in TAA, the DEGs from TAA overlapped with 1434 MRGs identified in the MsigDB database. As shown in Figure 1C, a total of 50 DE-MRGs in TAA were screened, including 32 upregulated and 18 downregulated genes. Detailed information on the DE-MRGs can be found in Table S2. The potential biological role of DE-MRGs specific for TAA vs. the control was determined via functional enrichment analysis using the Database for Annotation, Visualization, and Integrated Discovery (DAVID) database. In TAA, GO enrichment analysis showed that the DE-MRGs were mainly enriched in the mitochondria-mediated apoptosis pathway (Figure 1D). It was further found that the top 20 DE-MRGs were significantly different in TAA, and their contents were visualized using boxplots (Figure 1E).

### 3.2. Mitochondrial Dysfunction of Immune Cells in TAA Based on snRNA-seq

**Figure 2:**
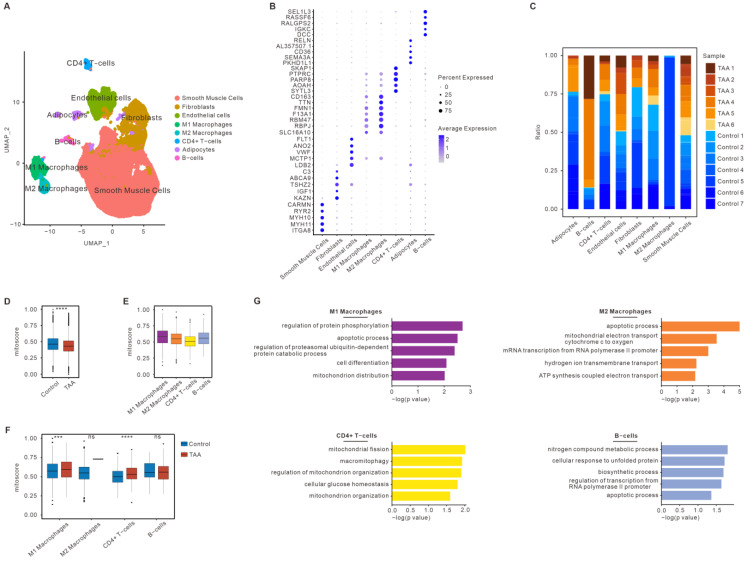
Mitochondrial dysfunction of immune cells in TAA based on snRNA-seq. (**A**) UMAP shows the annotation and color codes for the major cell types of all aorta cells from human TAA patients (32,277 cells, n = 6 patients) and a normal thoracic aorta (39,346 cells; n = 7 patients). Major cell types are defined using canonical lineage markers. (**B**) Dot plots of the specific cell type marker genes in TAA. (**C**) Fractions of the major cell types in each dataset of an aneurysmal aorta and a corresponding normal aorta of TAA. (**D**) Boxplots of the mitoscore between the TAA and control groups. (**E**) Boxplots of the mitoscore of the immune cell populations in TAA. (**F**) Boxplots of the mitoscore of the immune cell populations between the TAA and control groups. (**G**) Gene ontology enrichment analysis of the biological processes of the DE-MRGs in immune cells. ns: no significant difference. * *p* < 0.05; *** *p* < 0.001; **** *p* < 0.0001.

To further investigate the mitochondrial dysfunction in different cell populations, human snRNA-seq datasets were collected for TAA (GSE207784). After alignment and quality control (Figure S1), the GSE207784 dataset included 71,623 cells, comprising 39,346 cells from non-diseased thoracic aortic wall tissues (normal TA, *n* = 7) and 32,277 cells from aneurysmal thoracic aortic wall tissues (TAA, n = 6). The population structure of these human cells was analyzed and annotated according to acknowledged molecular markers. In TAA, the core cells were partitioned into 22 clusters with UMAP, with eight major cell types being identified. All cell types were classified as vascular wall cells (smooth muscle cells (SMCs), endothelial cells (ECs), and fibroblasts (FBs)), myeloid immune cells (M1/M2 macrophages), lymphocytes (CD4 + T cells and B cells), and others (adipocytes) (Figure 2A). The expression of these marker genes for each cell type was visualized using bubble plots (Figure 2B). As confirmed by quantification of the cellular components of each sample, there were significantly higher percentages of B cells. EC and SMC did not see significant changes compared with the normal group. Lower percentages of the other cells compared to the corresponding normal group were found in TAA, in which M2 macrophages were almost absent (Figure 2C). Using the above-mentioned methods, the mitoscores were calculated for each population of cells in each sample. First, analysis of the snRNA-seq datasets also revealed a decreased mitoscore in the TAA specimens compared to the control specimens (Figure 2D). The results of the above analysis are consistent with those of the bulk RNA-seq analysis. These results indicate that mitochondrial dysfunction is involved in the pathogenesis of TAA. In various immune cells of TAA, M1 macrophages showed the highest mitoscore among various immune cells, while CD4 + T showed the lowest mitoscore (Figure 2E). Furthermore, compared to the normal group, the mitoscore of M2 macrophages and B cells were no different in the TAA group, and the mitoscore of M1 macrophages and CD4 + T cells were increased (Figure 2F). DE-MRG analysis was performed on the immune cells. GO enrichment analysis indicated that the DE-MRGs were mainly enriched in the mitochondria-mediated apoptosis pathway for M1/M2 macrophages and B cells from patients with TAA, whereas DE-MRGs associated with mitochondrial quality control, such as mitochondrial fission, were found within CD4 + T cells (Figure 2G). These findings suggest that extensive mitochondrial function alters the immune cells of TAA.

### 3.3. Construction of a Diagnostic Model for TAA

**Figure 3:**
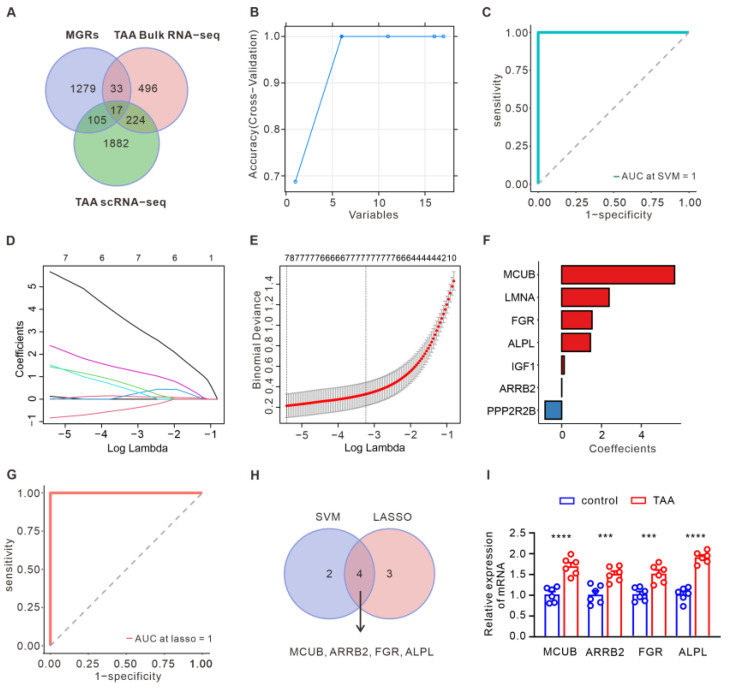
Construction of the diagnostic model for TAA. (**A**) Venn plot of the overlapped DE-MRGs in TAA from bulk RNA-seq and snRNA-seq analyses. (**B**) SVM-RFE algorithm of the variation curve of gene cross-validation error. (**C**) ROC curves of the AUC values of the SVM-RFE model. (**D**) Diagnostic model construction using a least absolute shrinkage and selection operator (LASSO) Cox regression model. (**E**) Coefficient distribution plots to select the optimum lambda value. (**F**) Bar charts of the selected genes and their coefficients. (**G**) ROC curves of the AUC values of the LASSO regression model. (**H**) Venn plots of the overlapped candidate genes from the SVM-RFE and LASSO regression models. (**I**) qRT-PCR was performed to detect the expression of *MCUB*, *ARRB2*, *FGR*, and *ALPL* in aortic aneurysm tissue of a TAA mouse model (*n* = 6) (data are presented as the mean ± SEM, *t*-test; *** *p* < 0.001; **** *p* < 0.0001).

Differential genes (|FC| > 1.5, FDR < 0.05) were obtained through differential analysis of snRNA-seq datasets in TAA. There were 2228 DEGs in total (1149 upregulated and 1079 downregulated) identified between the TAA and control groups (Figure S2 and Table S3). As shown in Figure 3A, 17 DE-MRGs were identified by overlapping the DE-MRGs from bulk RNA-seq and snRNA-seq analyses (Table 1). These 17 genes were subjected to create the diagnostic model, and two machine learning algorithms were used to identify the significant key DE-MRGs in TAA. Six candidate genes (*MCUB*, *ARRB2*, *ALPL*, *FGR*, *MAOA*, and *NAMPT*) were screened and validated in TAA based on SVM-RFE (Figure 3B). To minimize model overfitting, LASSO regression was applied to construct the prognostic model. Thus, a mitochondria-related prognostic signature with seven MRGs (*MCUB*, *LMNA*, *FGR*, *ALPL*, *IGF1*, *ARRB2*, and *PPP2R2B*) was established (Figure 3D–F). ROC was applied to evaluate the predictive classification efficiencies of the two models, as shown in Figure 3C,G. By overlaying the analyzed results of the two algorithms, key genes (*MCUB*, *ARRB2*, *FGR*, and *ALPL*) were identified as key MGRs for the discrimination of TAA and control specimens (Figure 3H). For further verification, mouse models of TAA were used to detect the content of the above genes in aortic aneurysm tissue using qRT-PCR, and consistent results were obtained (Figure 3I).

### 3.4. Analysis of the Key DE-MRGs and Metabolic-Related Genes

**Figure 4:**
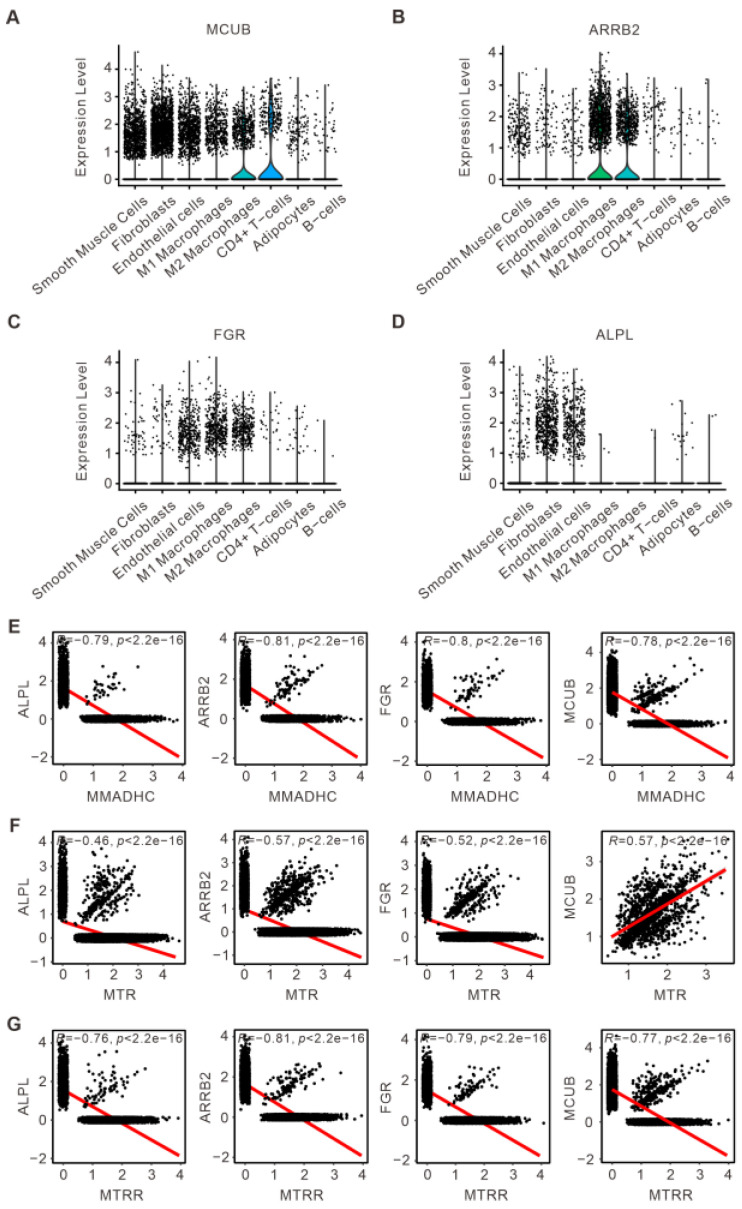
Analysis of mitochondria-related diagnostic genes and metabolism-related genes. (**A**–**D**) Violin diagram showing the expression of *MCUB*, *ARRB2*, *FGR*, and *ALPL* among the distinct cell subpopulations. (**E**–**G**) Pearson’s correlations between the mitochondria-related diagnosis gene expression and the genes associated with cobalamin and methionine metabolism, including *MMADHC*, *MTR*, and *MTRR*.

The cellular origin of the key MRGs mentioned above was further determined. In TAA, *MCUB* is widely present in various cells of the thoracic aorta wall, mainly in smooth muscle cells, endothelial cells, and macrophages, and less in B cells, CD4+ cells, and adipocytes (Figure 4A). *ARRB2* and *FGR* mainly exist in M1/M2 macrophages (Figure 4B,C). *ALPL* mainly exists in fibroblasts and endothelial cells (Figure 4D). Further investigation of the relationship between the key DE-MRGs and metabolic-related genes from the GeneCard database was performed using Pearson correlation analysis. We found three metabolism-related genes, including *MMADHC*, *MTR*, and *MTRR*, which are involved in the synthesis of cobalamin and methionine in cells. As shown in Figure 4E–G, *MCUB* and *MTR* are significantly positively correlated, while the other key MRGs are negatively correlated with these three metabolism-related genes. In summary, the above results indicate that the four key mitochondria-related genes identified in this study are distributed differently in cells and may affect the metabolism of methionine, thus participating in the progression of TAA.

## 4. Discussion

Mitochondria, as metabolic hubs regulating cellular energy, apoptosis, and metabolism, are promising diagnostic and therapeutic targets for cardiovascular disease management. While mitochondrial dysfunction VSMCs are known to contribute to TAA progression [12], alterations of mitochondrial function in immune cells and their role in TAA remain largely unexplored. Our findings revealed widespread mitochondrial dysfunction in immune cells associated with TAA, as well as altered mitochondrial functions and distinct mitochondrial-related genes (MRGs) linked to the disease. This study provides foundational insights into mitochondrial function in immune cells and suggests potential therapeutic targets for TAA.

Our analysis of bulk RNA-seq and single-nuclear RNA-seq (snRNA-seq) datasets, both sourced from the Genome Sequence Archive and NCBI-GEO, consistently demonstrated altered mitochondrial function in TAA. Bulk RNA-seq analysis showed differentially expressed MRGs (DE-MRGs) primarily involved in mitochondria-mediated apoptosis. In the single-nuclear dataset, four immune cell types were identified, with M1 macrophages exhibiting pronounced changes in the apoptosis pathway, while CD4 + T cells showed enhanced mitochondrial quality control. Though no significant mitochondrial dysfunction was detected in M2 macrophages or B cells, DE-MRGs were enriched in apoptosis-related processes. Only some immune cells were identified. The possible reasons for these are as follows: (1) GSE207784 is frozen human aortic tissue for high-quality single-nuclei analysis (this method of analysis results in fewer immune cells being obtained than single-cell RNA-seq) [18,19]; (2) a lower abundance of certain immune cells in diseased tissue; (3) varied hemodynamic effects and pathogenesis across arterial regions. These findings indicate that TAA-related immune cell mitochondrial dysfunction may arise through multiple mechanisms. Future studies aimed at modulating the mitochondrial function across cell types could inform precise TAA therapies.

We also identified key MRGs involved in TAA: *MCUB*, *ARRB2*, *FGR*, and *ALPL*. *MCUB*, a subunit of the mitochondrial calcium uniporter complex, regulates calcium transport into mitochondria, modulating energy metabolism and cell death [20]. Its expression increases following acute myocardial ischemia, offering cardioprotection by limiting mitochondrial calcium overload. It is also involved in maintaining cardiac systolic function in permanent myocardial infarction and severe heart failure [21,22,23]. *ARRB2*, a multivalent adapter protein, switches the *GPCR* from a G-protein signaling mode that transmits short-lived signals from the plasma membrane via small-molecule second messengers and ion channels to a beta-arrestin signaling mode [24]. *ARRB2* also participates in the regulation of body fluid and blood pressure, induces hypertrophy of cardiomyocytes through angiotensin II receptor type 1, and causes the death of cardiomyocytes after ischemia–reperfusion injury through the PI3K-Akt pathway [25,26]. Recent studies have shown that *ARRB2* promotes the ubiquitination of immune-responsive gene 1, which, in turn, promotes the release of mitochondrial reactive oxygen species and the production of M1-type macrophages [27]. FGR, a member of the Src family of protein tyrosine kinases, is predominantly expressed in various cell types of hematopoietic lineage, i.e., leucocytes and platelets, and is involved in the regulation of immune responses. It has been found to mediate leukocyte activation and migration through its interaction with integrins. ROS in tissues can also activate this kinase and promote the polarization of macrophages to regulate the inflammatory response [28,29]. *ALPL* encodes a tissue non-specific alkaline phosphatase, a key enzyme in ectopic calcification, whose overactivity in VSMCs and macrophages promotes aortic valve calcification, and cardiac fibrosis through pathways like TGF-β1/Smads, ERK1/2, and p53 [30,31,32,33]. Consistent with prior studies, *ARRB2* and *FGR* play roles mainly in macrophages, while *ALPL* is more active in fibroblasts and VSMCs. Although direct evidence linking these genes to TAA is limited, the above evidence points in this direction, and their roles in cardiovascular health suggest their potential as therapeutic targets.

It has been found that methionine metabolism dysfunction can affect the tricarboxylic acid cycle in mitochondria and thus affect its function [34]. Our study also found that these key MRGs are related to the methionine metabolism. Among them, *MMADHC* encodes a mitochondrial protein crucial in early vitamin B12 metabolism [35]. *MTRR* functions in the synthesis of methionine by regenerating methionine synthase to a functional state [36]. *MTR*, also known as cobalamin-dependent methionine synthase, catalyzes the final step in methionine biosynthesis. The processing of cobalamin in the cytosol occurs in a multiprotein complex composed of at least *MMADHC*, *MTRR*, and *MTR*, which can contribute to the safe and efficient shuttling of cobalamin toward *MTR* to produce methionine [37]. These genes are involved in the synthesis of cobalamin (vitamin B12) and methionine. Disruptions in cysteine metabolism may lead to abnormal homocysteine and glutathione production, as well as abnormal DNA methylation, contributing to TAA formation [38,39]. These findings support MRGs as potential diagnostic and therapeutic targets for TAA, warranting further validation studies.

Despite its contributions, our study has limitations. First, all data were derived from public datasets, and statistical adjustments cannot fully eliminate batch effects. These snRNA-seq datasets have limited samples and cells, possibly introducing variability due to human sample heterogeneity. Additionally, while key genes were validated in mouse models, their specific molecular mechanisms in human TAA remain uncharacterized. While there are similarities between species, animal models have not been fully effective in studying the pathological mechanisms of human aneurysm disease. Therefore, additional research is necessary, using clinical cohorts for further validation.

## 5. Conclusions

In conclusion, our integrative bioinformatics analysis revealed significant mitochondrial dysfunction in TAA. We identified four key mitochondrial-related genes—*MCUB*, *ARRB2*, *FGR*, and *ALPL*—that show promise as potential diagnostic and therapeutic targets for TAA. However, further experimental and clinical studies are necessary to clarify the roles of these genes in TAA pathogenesis and their potential in clinical applications.

## Figures and Tables

**Table 1:** Differentially expressed MRGs in TAA.

Gene Symbol		
*LMNA*	*ARRB2*	*ACADL*
*MCL1*	*SREBF1*	*DCN*
*PPP2R2B*	*MRPS6*	*MAOA*
*IL6*	*PLN*	*IGF1*
*ALPL*	*TFRC*	*NAMPT*
*FGR*	*MCUB*	

## Data Availability

The data used to support the findings of this study are available from the corresponding author upon request.

## Supplementary Materials

The following supporting information can be downloaded at https://www.mdpi.com/article/10.3390/bioengineering12020197/s1: Figure S1: Single-nuclear RNA-sequencing analysis of TAA.; Figure S2: snRNA-seq summary of the DEGs in TAA; Table S1: DEGs_TAA_vs_Control_bulk RNA-seq; Table S2: DE-MRGs_TAA_bulk RNA-seq; Table S3: DEGs_TAA_snRNA-seq; Table S4: qRT-PCR primers used for aortic expression analyses in a mouse model.
