# Supplementary material for: Utilizing Integrated Bioinformatics Analysis to Explore Potential Alterations in Mitochondrial Function Within Immune Cells Associated with Thoracic Aortic Aneurysms
Source: Bioengineering (Basel). 2025 Feb 17;12(2):197. doi: 10.3390/bioengineering12020197 (PMC11852063; doi:10.3390/bioengineering12020197)
Supplement: Supplementary file 1 [file bioengineering-12-00197-s001.zip › Supplementary Materials.pdf]

Supplementary Materials

Utilizing integrated bioinformatics analysis to explore potential alterations in mitochondrial function within immune cells associated with thoracic aortic aneurysms

Chang Guan[1], Si-xu Chen[2], Chun-ling Huang[2], Yi-peng Du[1], Kai-hao Wang[1], Pei-xin Li[1], Shenrong Liu[1], Zhao-yu Liu[2], Zheng Huang[1]

1. Department of Cardiology, the First Affiliated Hospital of Guangzhou Medical University, Guangzhou 510120, Guangdong, China.

2. Medical Research Center, Guangdong Provincial Key Laboratory of Malignant Tumor Epigenetics and Gene Regulation, Sun Yat-Sen Memorial Hospital, Sun Yat-Sen University, Guangzhou 510120, China

Supplemental Figures

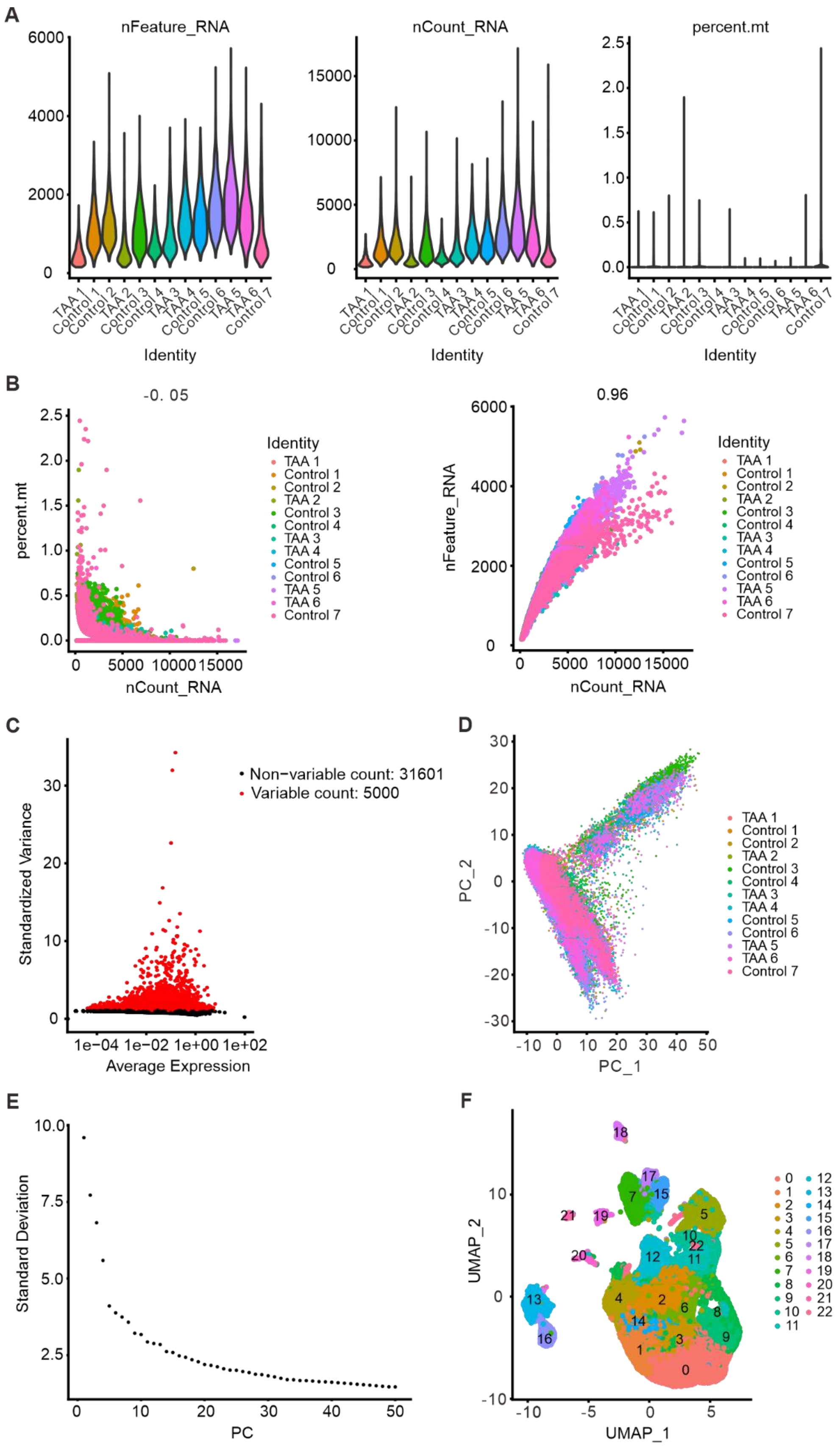

Figure S1. Single nuclear RNA-sequencing analysis of TAA. (A) The violin plots of the genes (features), counts, and mitochondrial gene percentage of each sample filtering after. (B) Scatter plots of the correlation between nCount_RNA and percent.mt, as well as between nCount_RNA and nFeature_RNA. (C) Scatter plots of HVGs were colored in red. (D-E) Scatter plots and JackStrawPlots of the PC selection. (F) UMAP of the distribution of the PCs.

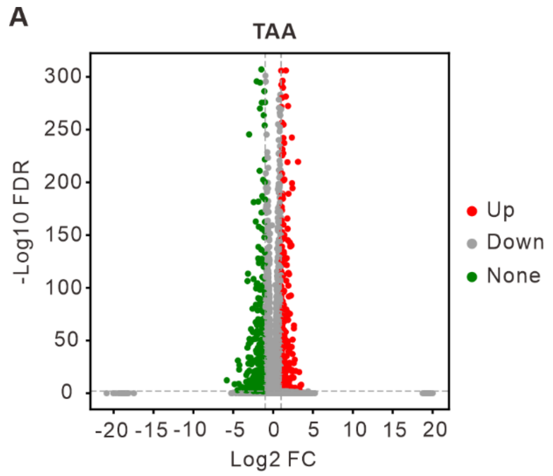

Figure S2. snRNA-seq summarizes the DEGs in TAA

(A) Volcano plot of DEGs in TAA.

Table S4. qRT-PCR primers used for aortic expression analyses in mouse

gene	sense	antisense
MCUB	CCACACCCCAGGTTTTATGTATG	ATGGCAGAGTGAGGGTTACCA
ARRB2	GGCAAGCGCGACTTTGTAG	GTGAGGGTCACGAACACTTTC
FGR	CGGCTGAAGAACGCTATTACC	GGGCGACGAATATGGTCACTC
ALPL	CCAACTCTTTTGTGCCAGAGA	GGCTACATTGGTGTTGAGCTTTT
